# Visualization of deep choroidal vasculatures and measurement of choroidal vascular density: a swept-source optical coherence tomography angiography approach

**DOI:** 10.1186/s12886-020-01591-x

**Published:** 2020-08-05

**Authors:** Erqian Wang, Xinyu Zhao, Jingyuan Yang, Youxin Chen

**Affiliations:** 1 Key Laboratory of Ocular Fundus Disease, Chinese Academy of Medical Sciences, Beijing, 100730 China; 2 Department of Ophthalmology, Peking Union Medical College Hospital, No.1 Dongshuaifuyuan, Dongcheng District, Beijing, 100730 China

**Keywords:** Optical coherence tomography angiography, Swept-source, Choroidal vascular density, Subfoveal choroidal thickness, Central serous chorioretinopathy

## Abstract

**Background:**

To investigate swept-source optical coherence tomography angiography (SS-OCTA) for deep choroid visualization and choroidal vascular density (CVD) measurement.

**Methods:**

Healthy subjects and central serous chorioretinopathy (CSC) patients were recruited for macular SS-OCTA scans. We evaluated OCTA images at various depths to determine an optimal depth for visualizing choroidal vasculatures. We measured CVD with binarized OCTA images at the optimal depth. In healthy subjects, CVD was studied for its correlation with age, axial length (AL), and subfoveal choroidal thickness (SCT). In CSC eyes, CVD was compared with matched controls.

**Results:**

Ninety-one healthy eyes and 22 CSC eyes were included. SS-OCTA could display deep choroidal vasculatures as dark signals, with 100 μm beneath BM as the optimal depth. In healthy subjects, the CVD at 100 μm beneath BM (56.5 ± 10.9%) was significantly correlated with SCT (*P* = 0.004) but not with age (*P* = 0.49) or AL (*P* = 0.72). In CSC eyes, the CVD at 100 μm beneath BM (62.3 ± 6.6%) was larger than that in 22 matched controls (54.1 ± 8.0%) (*P* = 0.001). The difference remained statistically significant after adjusting for SCT (*P* = 0.02).

**Conclusions:**

SS-OCTA can be used for visualizing deep choroidal vasculatures. CVD measured by OCTA at 100 μm beneath BM is a useful parameter for quantifying choroidal vascular status.

## Background

Choroid, a layer of vascular tissue, plays an important role in physiological and pathological process of human eyes. Visualization of choroidal vasculature is helpful in improving our understanding in a number of retinal and choroidal diseases. The swept-source optical coherence tomography (SS-OCT) technique permits deep choroid penetration. En-face OCT images of the choroid are useful for intuitive visualization of choroidal vasculatures and quantitative assessment of choroidal vascular density (CVD) [1, 2]. Quantification of CVD in deep choroid by en-face SS-OCT have been reported in healthy eyes [3], diabetic retinopathy [4], age related macular degeneration, polypoidal choroidal vasculopathy, and central serous chorioretinopathy (CSC) [5].

Optical coherence tomography angiography (OCTA), widely used for non-invasive imaging of retinal and choriocapillary microvasculatures [6, 7], has long been doubted for its value in deep choroid imaging. According to a previous report by Diaz et al. [8], intact retinal pigment epithelium (RPE) was regarded as a barrier for reliable detection of choroidal blood flow with OCTA. One previous study employed RTVue OCTA device for the quantification of deep choroidal vasculatures [9]. However, they used a built-in algorithm which was normally used for measuring choriocapillary flow density and was highly unlikely to quantify deep vasculatures correctly. Choi et al. [10] described a prototype SS-OCTA device at 1060 nm wavelengths with a 400 kHz A-scan rate in imaging Sattler’s layer of choroid. However, their equipment was commercially unavailable for wide application. Wang et al. [11] explored the ability of a commercially available SS-OCTA device to visualize and quantify the inner, middle, and outer choroidal vasculatures in a small series of eyes. However, there are still several gaps in current literature. First, the optimal depth for CVD measurements with SS-OCTA has not been identified. Second, the CVD measured with SS-OCTA has not been analyzed for its correlation with demographic factors. Third, the CVD measured with SS-OCTA has not been applicated in chorioretinal diseases.

To fill these gaps, we evaluated OCTA images at various choroidal depths with en-face OCT as reference standard, determined an optimal depth for visualizing choroidal vasculatures with OCTA, measured CVD with binarized OCTA image at the optimal depth, studied the OCTA derived CVD for its correlation with demographic factors in healthy subjects, and tested its usefulness in a series of CSC eyes. The purpose of this study is to improve our understanding in SS-OCTA for its capability in displaying deep choroidal vasculatures and quantifying deep choroidal vascularity.

## Methods

Ethics committee of Peking Union Medical College Hospital approved this observational case series study. All participants gave informed consents, and we conducted the study in accordance with the ethical tenets of the Declaration of Helsinki.

### Subjects

Healthy volunteers between 18 and 70 years without known systematic or ocular diseases were recruited and screened with a series of ophthalmic examinations including best-corrected visual acuity (BCVA), intraocular eye pressure (IOP), slit-lamp biomicroscope, indirect ophthalmoscope, axial length (AL), and macular 12.0 mm radial SS-OCT scan (DRI OCT Triton plus, Topcon Corporation, Tokyo, Japan). Subfoveal choroidal thickness (SCT) was measured at the center fovea between outer edge of retinal pigment epithelium and choroidal-scleral interface. Subjects were excluded if they had any one of the following circumstances in one or both eyes: 1. BCVA of less than 20/20; 2. IOP of more than 21 mmHg or less than 10 mmHg; 3. suspected vitreoretinal, choroidal or optic nerve diseases detected by screening examinations; 4. media opacity resulting in poor-quality image; and 5. AL of more than 26.50 mm or less than 20.50 mm.

A prospective consecutive series of eyes with acute or chronic CSC were recruited as one test group. Acute CSC was defined as a recent (< 3 months) onset of subretinal fluid through a focal leakage without prior episodes. Chronic CSC was defined as prolonged (> 3 months) onset of symptoms with widespread pigment epithelium abnormalities, with or without subretinal fluid. We measured AL and SCT for all CSC eyes. A group of age-, gender-, and AL- matched healthy controls were selected from the above eligible healthy subjects.

### OCTA scans and image acquisitions

**Fig. 1:**
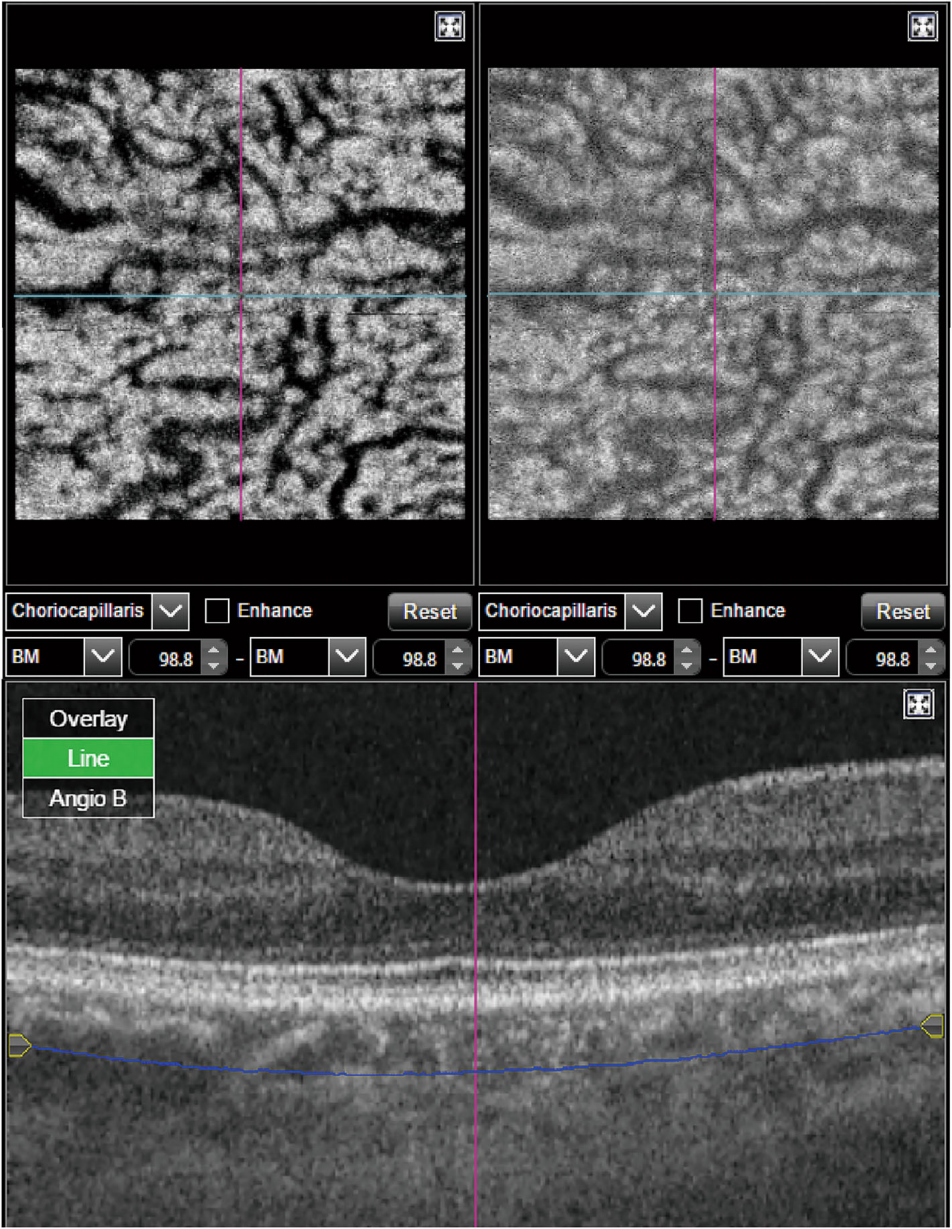
Simultaneous construction of OCTA and en-face OCT image pairs based on one single OCTA scan. (Top left) The OCTA image of a single-pixel slab at 98.8 μm beneath Bruch membrane (BM). (Top right) The en-face OCT image of the same slab. The OCTA and en-face OCT images showed similar pattern, in which dark stripes corresponded to the choroidal vessels. (Bottom) The position of the slab is shown by the arc line parallel to the BM

A 3.0 × 3.0 mm macular SS-OCTA scan (DRI OCT Triton plus, Topcon Corporation, Tokyo, Japan) were performed in both eyes of all eligible healthy volunteers and in all CSC eyes. The OCTA device worked at 1050 nm wavelength with a 100 kHz A-scan rate and a repeated scan count of 4 times. Each OCTA scan was comprised of 320 × 320 A-scans with an axial resolution of 8 μm. The built-in Topcon software allowed simultaneous construction of 300 × 300-pixel depth-resolved OCTA and en-face OCT images based on one single scan. Therefore, the OCTA and en-face OCT images were generated from exactly the same area and there was no need for manual registration. OCTA and en-face OCT image pairs were built at every 2.6 μm beneath between Bruch membrane (BM) by designating the same value for the starting and ending depth of each single-pixel slab (Fig. 1) [12]. The program automatically gave a score evaluating the sharpness and contrast of image series. Eyes with image score < 50 were excluded. Two investigators independently evaluated image artefacts for all OCTA image series. The investigators also confirmed that the BM of all subjects were recognized appropriately and there was no false segmentation due to pigment epithelium detachment or pachychoroid. Finally, only eyes without banding, blink, vessel doubling, stretching, out of window, crisscross, or ≥ 2 motion artefacts which were wider than primary branch vein diameter were included for further analysis [13, 14].

### Evaluation of Choroidal vasculature visualization at various depths and determination of the optimal depth

For healthy subjects, we collected choroidal OCTA and en-face OCT image pairs in two depth series, the fixed depth series with 50 μm intervals and the percentile depth series with 12.5% SCT intervals. In fixed depth series, because the axial resolution of OCTA scan was 2.6 μm, the actual depths for image collection were 49.4 μm, 98.8 μm, 150.8 μm, 200.2 μm, 249.6 μm, 299.0 μm, 351.0 μm, 400.4 μm, 449.8 μm, 499.2 μm, and 551.2 μm beneath BM. In percentile depth series, the actual depths for image collection in each eye were set as close to 12.5, 25.0, 37.5, 50.0, 62.5, 75.0, and 87.5% of its SCT beneath BM as possible.

We evaluated healthy subjects for OCTA images at all selected depths with en-face OCT images as reference standard. First, three authors (E.W., X.Z., and J.Y.) independently assessed original OCTA images, resolved disagreement if there were any, and calculated the proportion of eyes with appropriate choroidal vessel display at each depth. Second, we binarized OCTA and en-face OCT image pairs and calculated the similarity between a binarized OCTA image and its en-face OCT counterpart, which was defined as the percentage of matched pixels in the total number of pixels compared. Image binarization was done with Otsu’s method which calculated the optimum threshold for image binarization by minimizing intra-class variance [15] using ImageJ software by Wayne Rasband (National Institutes of Health, Bethesda, Maryland, USA; available at http://rsb.info.nih.gov/ij/index.html). Image comparison and similarity calculation were made with an algorithm developed by MATLAB software (MathWorks, Inc., Natick, MA, USA). Based on the above qualitative assessment and quantitative comparison, we determined an optimal depth, where OCTA images were most likely to display choroidal vessels appropriately and were most similar to its en-face OCT counterparts.

### Measurement of Choroidal vascular density at the optimal depth

**Fig. 2:**
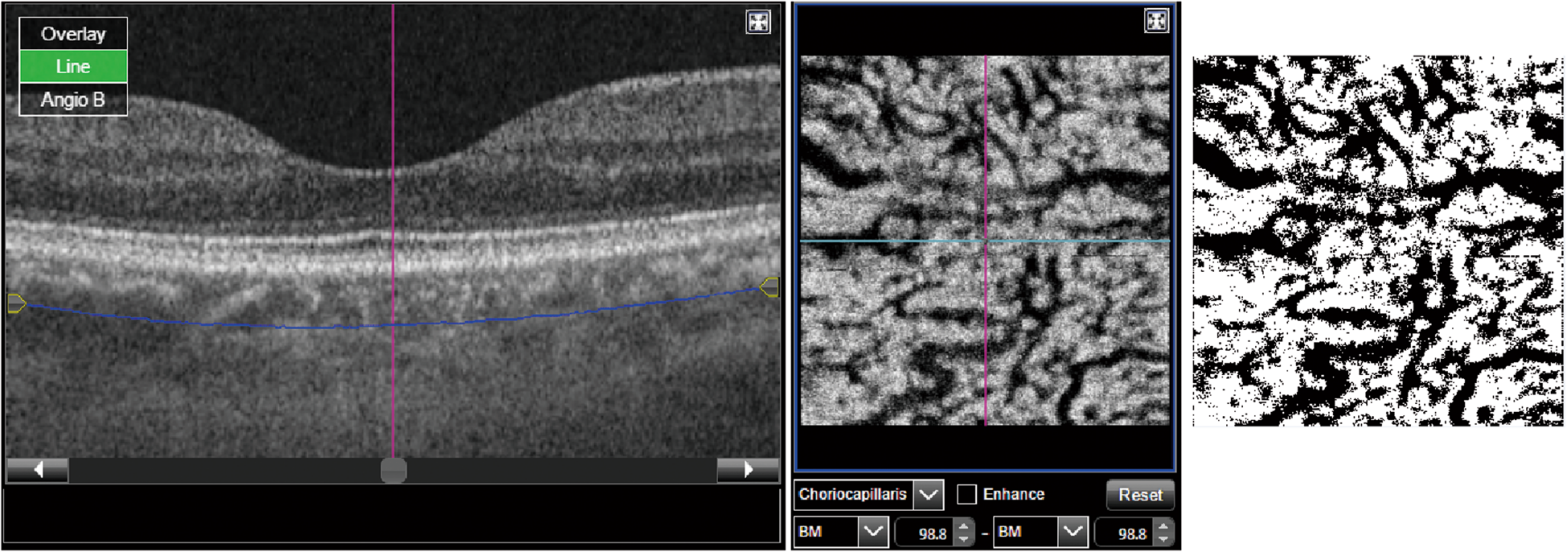
Measuring choroidal vascular density with OCTA. (Left) B-scan of the macular area. (Middle) Construction of a single-slab OCTA image at 98.8 μm beneath Bruch membrane. (Right) Binarization of the OCTA image. Choroidal vascular density was measured by the percentage of black pixels in the binarized image

We measured CVD of healthy and CSC eyes with binarized OCTA image at the optimal depth. An OCTA image at the optimal depth was binarized with Otsu’s method [15]. The black and white regions in binarized OCTA images were considered as vessel lumen and choroidal stroma, respectively. The CVD was defined as the proportion of black pixels from the total number of pixels in a binarized choroidal OCTA image [12] and was calculated using ImageJ software (Fig. 2).

### Statistical analysis

The statistical analysis was performed with SPSS software version 22.0 (IBM, Inc., Chicago, IL, USA). Kolmogrov-Smirnov method was used for normality test. Univariate Pearson correlation analysis and multivariate linear regression were performed to study the correlation between CVD and potential correlating factors including age, AL, and SCT. One-way analysis of variance (ANOVA) was used for the comparison of age, axial length, SCT, and CVD between CSC eyes and matched controls. Analysis of covariance (ANCOVA) was also used for the comparison of CVD between CSC eyes and matched controls, with SCT adjusted as covariates. A *P* value of less than 0.05 was considered statistically significant.

## Results

We included 116 eyes of 58 healthy participants for SS-OCTA scans. After excluding 2 eyes with blink artefact, 3 eyes with banding artefacts, 6 eyes with low image quality, and 14 eyes with at least two motion artefacts wider than primary branch vein diameter, we included 91 eyes from 51 healthy subjects (16 males and 35 females) for analysis. We also included 22 eyes with CSC (14 males and 8 females). All of the 5 acute CSC eyes and 13 of the 17 chronic CSC eyes had subretinal fluid overlying the scanned area. None of the CSC eyes had pachychoroid neovasculopathy. The characteristics of all included healthy eyes, CSC eyes and matched controls are shown in Table 1.

**Table 1:** Baseline characteristics of all included healthy eyes, CSC eyes, and matched controls

Characteristics	Healthy eyes (*n* = 91)	CSC eyes (*n* = 22)	Matched controls^*^ (*n* = 22)	*P*^†^
Age, years (mean ± SD)	39.4 ± 12.3	43.0 ± 8.4	42.9 ± 11.9	0.98
AL, mm (mean ± SD)	23.70 ± 1.18	22.82 ± 1.08	22.93 ± 0.98	0.70
SCT, μm (mean ± SD)	333.2 ± 94.2	547.3 ± 118.3	374.1 ± 101.7	**< 0.001**

Statistically significant *P* value is shown in bold

* Matched controls were selected from the included healthy eyes

† *P* values are from one-way analysis of variance (ANOVA) between CSC eyes and matched controls

*CSC* central serous chorioretinopathy, *AL* axial length, *SCT* subfoveal choroidal thickness, *SD* standard deviation

### Patterns of Choroidal OCTA images at various depths and the optimal depth for Choroidal vasculatures visualization

**Fig. 3:**
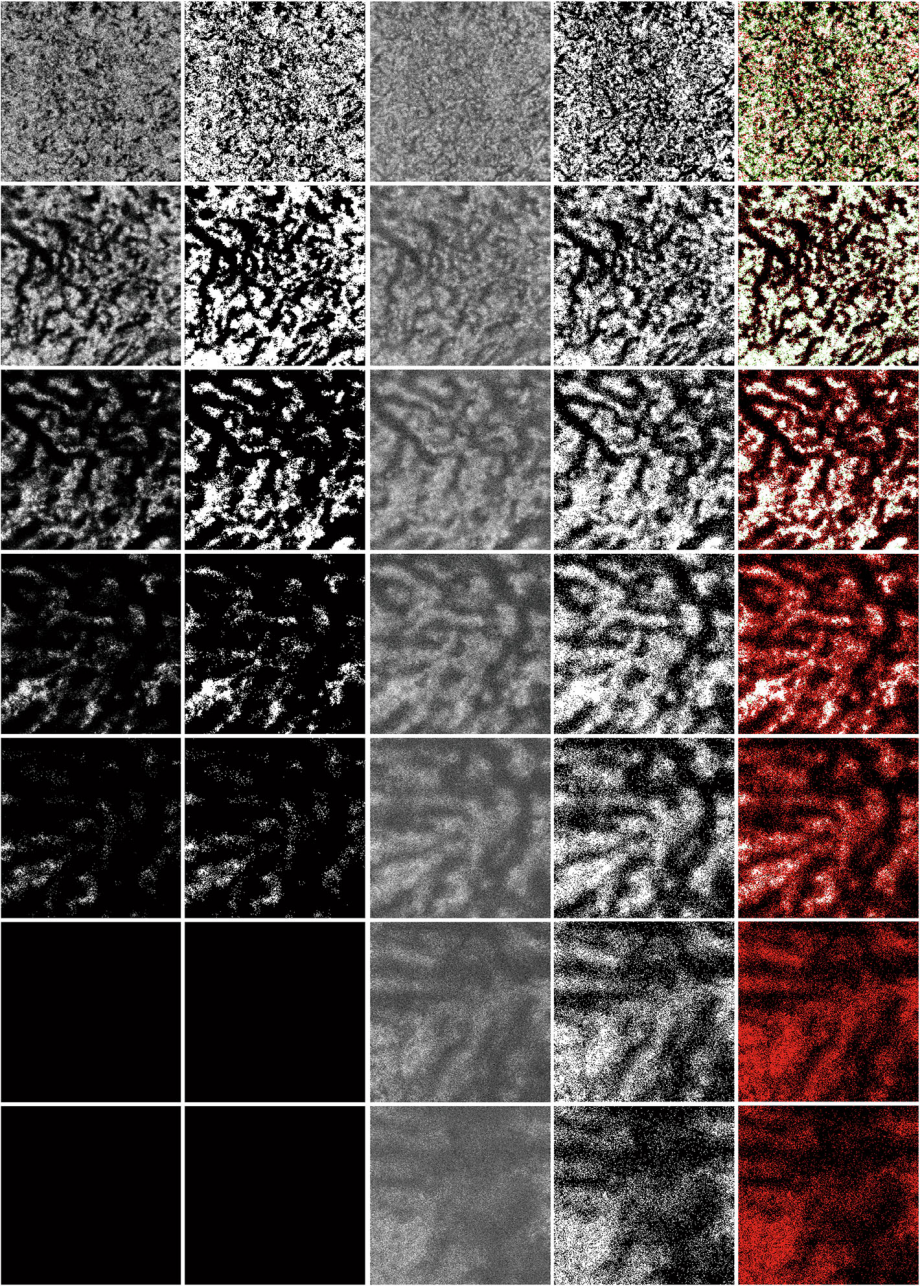
Representative OCTA and en-face OCT images at various choroidal depths. The SS-OCTA scan was performed in the right eye of a healthy subject in his 6th decade of life with central choroidal thickness of 378 μm. The five columns, from left to right, showed original OCTA images, binarized OCTA images, original en-face OCT images, binarized en-face OCT images, and comparison images. In comparison image, a pixel would be shown in white, black, green, and red, respectively, when the corresponding pixel was white in both OCTA and en-face OCT, black in both OCTA and en-face OCT, white in OCTA but black in en-face OCT, and black in OCTA but white in en-face OCT. The seven rows, from top to bottom, showed images at 50 μm, 100 μm, 150 μm, 200 μm, 250 μm, 300 μm, and 350 μm beneath Bruch membrane (BM). At 100 μm beneath BM, OCTA image displayed choroidal vessels as clearly as en-face OCT image, and the similarity between OCTA and its en-face OCT counterpart peaked at 82.0%

OCTA images at choriocapillaris exhibited granular pattern. As imaging plane went deep, OCTA images displayed choroidal vessels as dark stripes, which were similar to en-face OCT images. OCTA images at extremely deep level of choroid turned blurry or even black due to significant signal loss, most of which failed to display choroidal vessels as en-face OCT images did. Representative OCTA images, en-face OCT images, binarized images and image comparison in a subject with SCT of 378 μm are shown in Fig. 3.

The choroidal vascular display on OCTA images and image similarity between OCTA and en-face OCT images at fixed and percentile depths are shown in Table 2 and Table 3, respectively. The optimal depth in fixed depth series was 100 μm beneath BM where all OCTA images displayed choroidal vessels appropriately and similarity between OCTA and en-face OCT images peaked at 82.0 ± 2.6%. The optimal depth in percentile depth series was 25.0% of SCT beneath BM where 81 out of 91 (89.0%) OCTA images appropriately displayed choroidal vessels and similarity reached 79.6 ± 3.8%. In both series, OCTA images had limited capability in visualizing extremely deep choroidal vessels compared with en-face OCT images. Considering that the OCTA images at 25.0% of SCT beneath BM might not always display choroidal vessels and the minor variation in choroidal thickness measurement might result in a change of the slab localization, we selected 100 μm beneath BM as the optimal depth for measuring deep CVD with OCTA.

**Table 2:** The Number of OCTA Images Displaying Choroidal Vessels and Similarity Between OCTA and en-face OCT Images in Fixed Depth Series

Depth beneath BM (μm)	Number (%) of OCTA images displaying choroidal vessels	Similarity between OCTA and en-face OCT images (mean ± SD%)
50 (*n* = 91)	27 (29.7)	74.7 ± 4.2
**100 (*n*** **= 91)**	**91 (100.0)**	**82.0 ± 2.6**
150 (*n* = 89)	79 (88.8)	76.7 ± 6.5
200 (*n* = 86)	67 (77.9)	72.0 ± 7.2
250 (*n* = 69)	35 (51.5)	66.5 ± 6.2
300 (*n* = 58)	6 (10.3)	58.8 ± 6.4
350 (*n* = 44)	0 (0.0)	57.4 ± 7.2
400 (*n* = 23)	0 (0.0)	57.3 ± 5.7
450 (*n* = 7)	0 (0.0)	52.9 ± 1.5
500 (n = 4)	0 (0.0)	50.5 ± 0.5
550 (*n* = 1)	0 (0.0)	50.0

The optimal fixed depth with the largest percentage of OCTA images displaying choroidal vessels and the greatest similarity between OCTA and en-face OCT images is shown in bold

*BM* Bruch membrane, *OCTA* optical coherence tomography angiography, *OCT* optical coherence tomography

**Table 3:** The Number of OCTA Images Displaying Choroidal Vessels and Similarity Between OCTA and en-face OCT Images in Percentile Depth Series

Depth beneath BM (%SCT)	Number (%) of OCTA images displaying choroidal vessels	Similarity between OCTA and en-face OCT images (mean ± SD%)
12.5 (*n* = 91)	25 (27.5)	71.9 ± 6.5
**25.0 (*n*** **= 91)**	**81 (89.0)**	**79.6 ± 3.8**
37.5 (*n* = 91)	80 (87.9)	77.9 ± 5.4
50.0 (*n* = 91)	78 (85.7)	73.6 ± 8.3
62.5 (*n* = 91)	48 (52.8)	71.6 ± 9.2
75.0 (*n* = 91)	38 (41.8)	69.2 ± 10.6
87.5 (*n* = 91)	26 (28.6)	65.8 ± 12.0

The optimal percentile depth with the largest percentage of OCTA images displaying choroidal vessels and the greatest similarity between OCTA and en-face OCT images is shown in bold

*BM* Bruch membrane, *SCT* central choroidal thickness, *OCTA* optical coherence tomography angiography, *OCT* optical coherence tomography

### Choroidal vascular density and correlating factors in healthy subjects

Among the 91 healthy eyes, the mean CVD at 100 μm beneath BM was 56.5 ± 10.9%. Univariate analysis showed that CVD at 100 μm beneath BM was significantly correlated with SCT (*P* = 0.003) but not with age (*P* = 0.40) or AL (*P* = 0.67). Multivariate linear regression revealed that for every increase in SCT of 100 μm, CVD at 100 μm beneath BM increased by 3.9% (95% confidence interval 1.3 to 6.5%, *P* = 0.004). The CVD at 100 μm beneath BM had no significant correlation with age (*P* = 0.49) or AL (*P* = 0.72).

### Choroidal vascular density in CSC subjects and comparison with controls

**Fig. 4:**
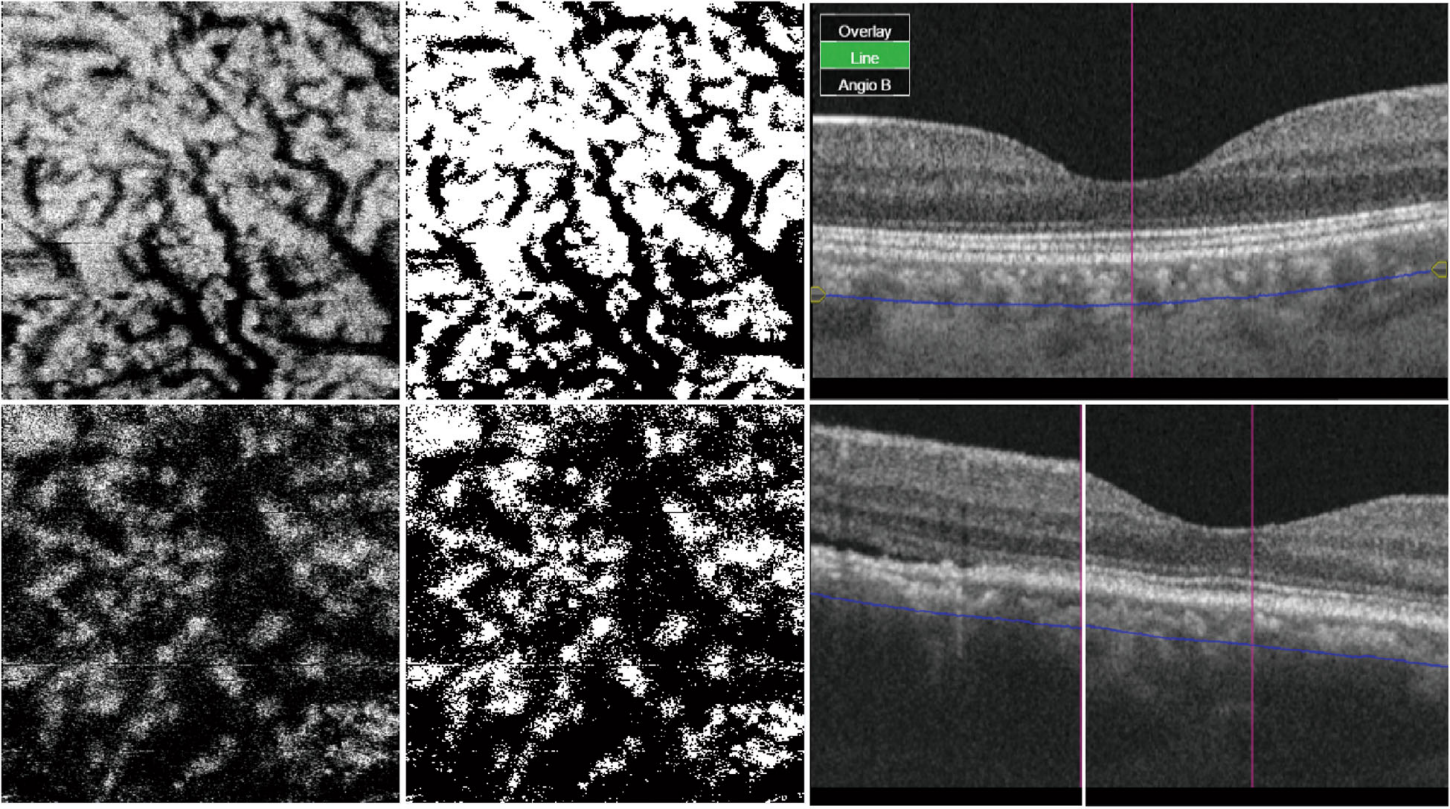
Representative OCTA images at 100 μm beneath Bruch membrane (BM) in healthy and central serous chorioretinopathy (CSC) eyes. (Top left) OCTA image of the right eye of a 31-year-old healthy female whose subfoveal choroidal thickness (SCT) was 288 μm and axial length (AL) was 23.40 mm. (Top middle) The binarized OCTA image of the healthy eye. (Top right) B-scan of the healthy eye with the arc line showing the imaging depth. (Bottom left) OCTA image of the right eye of a 41-year-old CSC male whose SCT was 507 μm and AL was 23.05 mm. (Bottom middle) The binarized OCTA image of the CSC eye. (Bottom right) OCT B-scan of the CSC eye with dilated choroidal vessels and subretinal fluid. Based on the OCTA image at 100 μm beneath BM, the choroidal vascular density was 58.2 and 71.5% in the healthy eye and the CSC eye, respectively

In one-way ANOVA, the CVD at 100 μm beneath BM was significantly increased in the 22 CSC eyes compared with 22 matched controls (62.3 ± 6.6% and 54.1 ± 8.0%, *P* = 0.001). In ANCOVA where SCT was adjusted as covariates, the CVD at 100 μm beneath BM in CSC eyes was still significantly larger than that in controls (*P* = 0.02). Representative OCTA images at 100 μm beneath BM in CSC and healthy eyes are shown in Fig. 4.

## Discussion

By systematically evaluating deep choroidal OCTA images in healthy and CSC eyes, the current study not only suggested the capability of SS-OCTA in displaying choroidal vessels, but also identified CVD at 100 μm beneath BM as a useful OCTA parameter. This parameter was stable across various age and axial length groups, and was valuable in reflecting choroidal vascular status.

There had been an impression that OCTA might not be suitable for imaging the choroid beneath choriocapillaris. According to Diaz et al., motion contrast signals produced bright vessel outlines only in areas with overlying RPE atrophy, and dark signals beneath intact RPE was deemed as absence of blood flow [8]. According to our study, within a range of choroidal depths, dark signals in OCTA images corresponded to vessel lumen in OCT B-scan, and deep choroidal vessels manifested as dark stripes in OCTA images. This phenomenon could be related with several underlying mechanisms including fringe washout effect, signal attenuation, and threshold masking. Fringe washout is the signal loss due to high blood flow velocity exceeding system imaging speed [16–18]. Attenuation is the signal blockage by RPE which reduces a significant portion of light entering choroid [13, 19]. Threshold masking is a process of signal removal in OCTA image building which ensures that only valid motion contrast signals were included and potential noises were excluded [19]. In our study, OCTA images and en-face OCT images displayed choroidal vessels as similar dark stripes within a range of choroidal depths. Therefore, the notion that OCTA cannot be used for deep choroid imaging might be biased and incomprehensive.

We identified 100 μm beneath BM as the optimal depth for the current SS-OCTA device in depicting choroidal vessels for two reasons. First, in all healthy subjects, OCTA images at 100 μm beneath BM were able to display choroidal vessels appropriately. Second, OCTA and en-face OCT images had greatest similarity at 100 μm beneath BM in almost all eyes with various choroidal thickness. The above facts suggested that capability of SS-OCTA in depicting choroidal vessels was likely to be determined by the intrinsic penetration of the SS-OCTA device, rather than the imaging preference of specific choroidal layers. Morphological studies from autopsy eyes showed that the thickness of BM and choriocapillaris ranged from 2.0 μm to 4.7 μm and from 6.5 μm to 9.8 μm, respectively [20]. In vivo investigation showed that subfoveal thickness of Sattler’s layer was 87 ± 56 μm [21]. We thereby inferred that the choroidal OCTA image at 100 μm beneath BM was likely to describe vasculatures around the boundary between Sattler’s and Haller’s layer.

Because deep choroidal vessels were displayed as dark stripes in OCTA images, the quantification of vascular density should be conducted by manual calculation of the proportion of dark signals instead of the automatic measurement of area of bright pixels generated by the built-in software. Chan et al. reported their OCTA measurements of CVD in deep choroid of CSC patients before and after photodynamic therapy (PDT). They agreed that the dark stripes in deep choroidal OCTA images reflected blood vessels. However, they measured CVD with a built-in algorithm which calculated the proportion of bright signals, normally used for measuring choriocapillary blood flow [9]. They reported a reduction in choroidal vessel diameter but an increase in vascular density after PDT, which was self-contradictory and inconsistent with a number of previous observations on PDT treatment [22, 23].

We revealed that CVD measured by SS-OCTA at 100 μm beneath BM is a useful parameter for quantifying choroidal vascular status. First, no manual calibration of SCT is needed in calculating CVD. The fixed depth, or 100 μm beneath BM, for image generation is likely to minimize potential measurement bias among different technicians. Second, this parameter was correlated with choroidal thickness and was relatively stable across various age and refraction groups. One previous study by Fujiwara et al. [3] utilized en-face OCT for the measurement of CVD at 50% SCT beneath BM and reported that CVD was significantly correlated with SCT but not with AL, which is similar to our findings. However, they reported that CVD measured with en-face OCT was negatively correlated with age, which might be due to relatively large sample size and broad sample age. Third, the value of this parameter in reflecting choroidal vascularity was also tested and verified in a series of CSC eyes. A plenty of studies have shown increased choroidal blood flow or dilated choroidal vessels in CSC patients [12, 24]. In our study, CVD measured by SS-OCTA at 100 μm beneath BM was increased in CSC eyes, consistent with previous findings. One may argue that the increased CVD value in CSC eyes might be related with thick choroid. To eliminate the potential influence from SCT, we compared CVD between groups after adjusting for SCT and the difference remained significant. One may also argue that the presence of subretinal fluid or pigment epithelium detachment may produce shadowing artifacts in OCTA images of underlying structures and may result in an overestimation of CVD in deep choroid. However, the round dark “shadow” produced by subretinal fluid was almost invisible in deep choroid, because there was mild, universal, and uniform signal loss in deep choroid. Based on the above considerations, we believe that CVD at 100 μm beneath BM measured by OCTA is a potentially valuable and convenient parameter in future studies.

Despite the potential value of SS-OCTA in visualizing choroidal vessels and measuring CVD at 100 μm beneath BM, we have to admit that the capability of SS-OCTA in visualizing deep choroidal vasculatures is limited in extremely deep choroid where signal attenuation is significant. Signal attenuation in OCTA images results in more dark signals than actual and thereby causes overestimation of CVD [11, 18].

There were several limitations in the current study. First, the healthy group included more females than males, although no significant difference of CVD was observed between males and females (*P* = 0.36). Second, the healthy group included only 91 subjects. The correlation between CVD and SCT as well as the stability of CVD among various age and refraction groups need further confirmation in a larger sample. Third, our result is only applicable to the current SS-OCTA device, and the capability of other OCTA devices in visualizing deep choroid and measuring CVD remains unknown. Fourth, we observed choroidal OCTA images at predetermined depths with 50 μm intervals instead of a consecutive series of depths. Studies with refined choroidal depth intervals might add to our current knowledge in SS-OCTA imaging of deep choroid. Fifth, choroidal imaging was limited in a 3.0 × 3.0 mm macular area. A broader imaging coverage might be needed in future studies.

## Conclusions

The current SS-OCTA device was useful in visualizing choroidal vasculatures within a range of choroidal depths. Notably, this SS-OCTA device was able to measure CVD at 100 μm beneath BM, which was a useful parameter in quantifying choroidal vascular status. The study improved our understanding in OCTA technique and expanded its application in deep choroid imaging.

## Data Availability

The datasets used and/or analysed during the current study are available from the corresponding author on reasonable request.

## Abbreviations

OCT: Optical coherence tomography
SS-OCT: Swept source optical coherence tomography
OCTA: Optical coherence tomography angiography
SS-OCTA: Swept source optical coherence tomography angiography
CVD: Choroidal vascular density
SCT: Subfoveal choroidal thickness
CSC: Central serous chorioretinopathy
RPE: Retinal pigment epithelium
BCVA: Best corrected visual acuity
IOP: Intraocular eye pressure
AL: Axial length
BM: Bruch membrane
